# Adapting CBT-E for the Middle East: addressing regional gaps in eating-disorder treatment

**DOI:** 10.3389/fpsyt.2026.1761708

**Published:** 2026-05-12

**Authors:** Carine El Khazen, Maya Sidani, Hala Abu Taha, Bernou Melisse

**Affiliations:** 1 American Center for Psychiatry and Neurology (ACPN), Dubai, United Arab Emirates; 2 American Center for Psychiatry and Neurology (ACPN), Abu Dhabi, United Arab Emirates; 3 Co-eur, Utrecht, Netherlands; 4 Department of Clinical Psychology, Utrecht University, Utrecht, Netherlands; 5 Department of Medical and Clinical Psychology, Tilburg University, Tilburg, Netherlands

**Keywords:** body image, cultural adaptation, eating disorders, emotion regulation, enhanced cognitive behavior therapy, family involvement, Middle East & North Africa (MENA), Ramadan

## Abstract

Eating disorders were regarded as Western conditions, yet recent evidence shows that they do occur across the Middle East and North Africa. Despite this, the region remains underserved: specialized services are scarce, awareness is limited, and mental health stigma persists. For many years, patients were misdiagnosed, treated only after medical crises, or managed with non-specialized, eclectic psychotherapeutic approaches not designed for eating disorders. Moreover, established treatments, developed and validated largely in Western contexts, were neither available in Arabic nor culturally adapted to local realities. This Perspective paper outlines the rationale and process of adapting and implementing enhanced cognitive behavioral therapy (CBT-E), the gold-standard treatment for adults with eating disorders, within the first specialized eating-disorders program established in the United Arab Emirates in 2017. Adaptation was shaped by systemic barriers, linguistic challenges, and sociocultural and religious considerations: Ramadan fasting, modesty norms, and family involvement. Therapists supported patients in maintaining regular eating patterns during Ramadan by integrating Islamic exemptions and spiritually meaningful alternatives, and modified body-image procedures, particularly exposure and clothing-related tasks, to respect modesty while preserving CBT-E’s mechanisms of change. Family participation was sometimes broadened beyond standard CBT-E to reflect norms of interdependence, and extended caregiving roles. Cultural tailoring may support more equitable implementation by improving acceptability and sustaining engagement, as observed in routine clinical practice and informed by internal service-level monitoring used to guide iterative refinement of care. The present paper offers a foundation for future adaptation efforts and underscores the need to expand evidence-based eating-disorder care across underserved regions.

## 1 Introduction

Eating disorders were long conceptualized as Western illnesses, thought to primarily affect white, affluent women in industrialized societies (1, 2). This assumption obscured their presence in non-Western regions, including the Middle East and North Africa (MENA), where recognition and treatment development lag (3, 4). Recent meta-analyses estimate lifetime prevalence at 6.1% and 12-month prevalence at 3.2% (4–6). These figures, derived primarily from reviews rather than large-scale epidemiological studies (4, 5), nonetheless highlight the significant burden of eating disorders across MENA populations.

Multiple factors contribute to vulnerability to eating disorders across the region. Rapid Westernization has reshaped daily life through increased exposure to Western media, appearance ideals, and dieting culture (1, 2, 5–12). Additional contributors include exposure to conflict and trauma, economic inequity in parts of the region, food insecurity, and pervasive weight stigma across families, schools, and healthcare systems. All delaying help-seeking (13–20) and contributing to the low prioritization of eating disorders (19, 20). Until recently, specialized eating-disorder services across MENA were non-existent (4). Following the first author’s relocation to the UAE in 2004, a clinical initiative to develop specialized outpatient eating-disorder care emerged amid minimal mental-health infrastructure and an unexpected volume of eating disorders cases seen in a general medical polyclinic. At that time, eating disorders were often unrecognized, misdiagnosed, or treated only after medical deterioration (21). Subsequently, the UAE’s first specialized eating-disorders program was established in 2017, constituting an initial effort to develop dedicated regional services. Enhanced Cognitive Behavioral Therapy (CBT-E) was adopted as the program’s core intervention because of its transdiagnostic nature, suitability for adolescents and adults, and supported by a manualized structure that facilitates training, fidelity, and dissemination (22–25). As an individual therapy (26), it offered a practical starting point in a region where family involvement can be complex.

Initially based in Dubai, later across the UAE, CBT-E was delivered by a multidisciplinary team, the program addressed systemic, religious, and cultural factors (e.g., fasting during Ramadan, modesty norms, and family participation). Fidelity to CBT-E was supported through structured training and ongoing supervision. All members of the team completed the CREDO web-centered CBT-E training program prior to delivering treatment. Between 2017 and 2020, clinicians participated in monthly, two-hour, case-based supervision with Dr Riccardo Dalle Grave. This was complemented by weekly supervision led by the first author from 2017 to the present. Clinicians also used CBT-E–specific therapist-rated fidelity tools, including the Therapist Self-Rated Components Checklists for Adults and Adolescents (27), alongside routine clinical outcome monitoring.

The present paper is offered as a Perspective rather than a service model or outcome report. Drawing on the implementation of CBT-E in the United Arab Emirates, it advances the argument that culturally informed fidelity, rather than rigid protocol adherence or unstructured modification, is essential for the ethical dissemination of evidence-based treatments beyond Western settings.

We acknowledge that this Perspective is grounded in the UAE context, which is socioeconomically distinct from other parts of the region. Nevertheless, access to mental-health care remains heterogeneous, eating-disorder services are scarce, and insurance coverage varies substantially across populations; conditions that parallel those found across much of the MENA region. Additionally, the adaptations described—Arabic language delivery, accommodation of modesty norms, abstinence from fasting during Ramadan, culturally normative family involvement, and optional religious practices—also address shared cultural and religious realities across Arab Muslim populations and are not contingent on socio-economic status (20). This approach is consistent with Fairburn’s emphasis that CBT-E is guided by individualized case formulation and allows for the flexible selection of CBT-E–consistent procedures to address maintaining mechanisms as clinically indicated (26).

While substantial heterogeneity across the MENA region must be taken into consideration, broader application in lower-resource or conflict-affected settings will require additional feasibility work beyond the scope of this paper.

## 2 Cultural and clinical adaptations of CBT-E in the Middle East

### 2.1 Linguistic and cultural adaptation

CBT-E reflects assumptions about autonomy, communication, and family roles that do not always align with collectivist Arab contexts. Applying the model in the region required sensitivity to the linguistic, cultural, and systemic factors influencing engagement and feasibility. Adaptation at the level of delivery format and linguistic framing was required to ensure comprehension, collaboration, and engagement. Language posed a barrier, as all CBT-E materials are available only in English despite Arabic being the shared language across 22 countries (26). Treatment was therefore delivered by fluently Arabic–English-speaking therapists. Translation was limited to language, metaphors, and psycho-educational examples, including body-image–related constructs, to ensure comprehension and therapeutic resonance. This was done without altering protocol procedures or mechanisms, consistent with established frameworks for culturally informed translation in Arab mental-health contexts (6). Without culturally grounded psychoeducation and examples, engagement can be undermined (28–30). Hence, adaptation was essential, given that cultural and religious traditions shape experiences of food, body, and autonomy (3, 31, 32). Therapists collaborated with patients to refine key terms and concepts for linguistic precision and cultural resonance ensuring emotional accuracy without altering the treatment’s therapeutic intent. All procedures were reviewed and approved by the Centre for Research on Eating Disorders at Oxford, developers of CBT-E (22, 25, 26).

### 2.2 Family involvement

Although CBT-E for adults is delivered individually, therapists may, with patient consent, involve a significant other to enhance adherence or address practical barriers (26). In our clinical setting, there may be an increased need to involve significant others in specific circumstances, when addressing culturally sensitive topics such as exemptions from fasting during Ramadan. This involvement remains selective and aligned with standard CBT-E principles (26).

Conversely, CBT-E for adolescents incorporates structured parental involvement. This involvement is collaborative rather than directive: parents participate only with the adolescent’s agreement and are guided to provide support without criticism or control (33, 34).

In the regional context, however, complete adolescent autonomy is less common (35, 36). Consequently, therapists may sometimes need to balance CBT-E’s collaborative ethos with the practical need for greater family participation. While parents may adopt a more active supportive role, this participation remains aligned with the interventions non-coercive principles (26).

Moreover, in some families, especially when parents are elderly or absent, older siblings may assume caregiving roles, making their involvement essential in place of parents, a pattern less common in Western contexts. Many young adults in the region continue to live with their parents, depend on them financially, and include them in health-related decisions (35, 37). Building on this context, elements of the adolescent CBT-E framework were applied selectively to young adults who were financially dependent, living at home, and developmentally positioned similarly to adolescents, with parental involvement introduced flexibly when it supported treatment delivery.

Across both adult and adolescent cases, these culturally sensitive adaptations may enhance engagement. Facilitated by CBT-E’s flexible and personalized structure, they were implemented within a collaborative stance ensuring that additional family participation supported rather than replaced the patient’s responsibility for change (26).

### 2.3 Ramadan and regular eating

In parallel with these cultural and family adaptations, there was also a need to tailor the delivery of some CBT-E behavioral procedures to ensure they remained feasible and clinically resonant. One key element is establishing regular eating, introduced in Stage 1. This involves three meals and two to three planned snacks spaced through the day, with no unplanned eating, this forms the behavioral foundation for later therapeutic work (26). However, Ramadan, one of the five pillars of Islam, is a sacred month of fasting from dawn until sunset. The fast is broken at sunset with the communal evening meal (iftar) and begins each day with a pre-dawn meal (suhoor); the month concludes with Eid al-Fitr (Qur’an 2:183–185). Fasting is both a religious duty and a strong social expectation, and those unable to fast may experience exclusion or shame. For individuals with eating disorders, however, fasting can trigger or perpetuate restrictive behaviors, binge eating, or compensatory cycles (38). Therefore, major clinical guidelines advise against it (39). Additionally sustained fasting also makes regular eating impossible and prevents CBT-E from being implemented as intended.

As life-threatening psychiatric illnesses with among the highest mortality rates (40, 41), eating disorders fall within the Qur’anic exemption from fasting for those who are ill (Qur’an 2:184). In consultation with religious authorities, it is confirmed that individuals with eating disorders are exempt from fasting during periods of illness. Patients and families are educated about this ruling, emphasizing that nourishment and treatment adherence align with Islamic principles of preserving life and health. Islamic jurisprudence recognizes alternative acts of worship for individuals unable to fast for medical reasons, ensuring continued spiritual engagement without compromising health (Qur’an 2:184). Spiritual alternatives to fasting are explored as ways of maintaining connection to faith while supporting treatment progress and facilitating adherence to fasting abstinence (Qur’an 2:184). Additionally, linking recovery behaviors to Islamic values of mercy (rahma) and stewardship of the body (amana) reduced self-criticism and supported recovery stability during Ramadan (Qur’an 6:141; Qur’an 2:195). Patients are encouraged to attend family or community iftar gatherings even when not fasting, to maintain connection and reduce feelings of exclusion. Specialized CBT-E dietitians work collaboratively with patients to provide flexible dietary guidelines or individualized meal plans when needed. This approach enables participation in family and community gatherings while preserving the therapeutic structure of regular eating. Therapists also guide patients in developing assertive yet respectful responses to questions or criticism from relatives or community members.

### 2.4 Alternatives to binge-eating

In Stage 1, patients learn strategies to manage urges to eat between planned meals. Therapists explain that these urges are time-limited and can be observed without judgment, while engaging in alternative activities chosen collaboratively. These activities are realistic, active, and enjoyable, such as hobbies or brief tasks. With practice, patients learn that urges fade without acting on them, strengthening confidence and self-efficacy (26). In the Middle Eastern context, these procedures are preserved while the range of optional alternatives may include religious and spiritual practices that serve similar regulatory functions. For some Muslim patients, prayer, Qur’an recitation, ablution (woudou), or rhythmic use of the rosary (masbaha) provide structured, soothing, and time-limited rituals that redirect attention from urges while fostering calm and reflection. These options are introduced collaboratively and offered alongside other strategies, ensuring they are experienced as authentic and non-coercive.

Similarly, cultural factors provided additional therapeutic entry points in the application of CBT-E Stage 3 “Events, Moods, and Eating”. In this module, proactive problem-solving is introduced as a strategy to anticipate and reduce exposure to situations likely to trigger negative emotions and subsequent eating-disorder behaviors (26). The same optional activities, including religious or spiritual practices when relevant, may be incorporated preventively.

The incorporation of religious or spiritual practices within emotion-regulation work did not constitute a structural modification of the treatment protocol. Consistent with the model, therapists did not prescribe specific strategies; alternatives were selected collaboratively and individualized (26). Religious practices were introduced only when patient-initiated, optional, and clinically appropriate, serving the same regulatory functions as other CBT-E–consistent strategies. Accordingly, these adaptations concerned delivery and contextual resonance rather than core therapeutic components, preserving CBT-E’s mechanisms and structure while enhancing cultural relevance. Across adaptations, the therapeutic goal remained unchanged: to weaken the association between mood and eating and support lasting control over eating through personally valued strategies (26).

### 2.5 Body image and modesty

Importantly, the module that required the most extensive cultural adaptation was body image. Within Stage 3, the “Body Image Module” treatment targets the core underlying psychopathology of eating disorders: the over-evaluation of shape and weight and their control. This is when one’s self-worth becomes predominantly and largely dependent on controlling one’s body shape and weight. The therapeutic approach relies on a two-pronged strategy. First, it aims to broaden self-evaluation by helping patients re-invest in alternative domains such as relationships, work, values, or spirituality. Second, it addresses the behavioral expressions of the over-evaluation, including body checking, body avoidance, and “feeling fat” (26). Of the three expressions of the over-evaluation of shape and weight only “body avoidance” is substantially shaped by local cultural and religious norms. The other maintaining behaviors can be addressed using standard CBT-E procedures.

Addressing body avoidance requires gradual exposure to situations typically avoided because of discomfort with seeing one’s own body or being seen by others, such as wearing fitted clothing, swimsuits, or engaging in activities that increase body visibility. Together, these procedures weaken the dominance of shape and weight in self-worth and foster a more balanced self-evaluation (26). In many Arab and Muslim societies, expectations of modesty, rooted in religion and social convention, govern dress, self-presentation, and bodily visibility (42). This derives from the concept of “awra”, which delineates parts of the body that should remain covered to preserve modesty, commonly the entire body except the face and hands for women (Qur’an 24:30–31; Qur’an 33:59). In this context, many women are veiled, wearing the abaya (a long, loose outer garment) and covering their heads or hair as a reflection of modesty and faith. Modesty principles typically involve loose, full-length clothing that limits bodily exposure including among women who do not wear the abaya but nonetheless adhere to religiously informed norms of dress (42). Such norms influence how patients engage in exposure-based procedures that involve observing, or being observed, physically.

Exposure-based procedures in CBT-E, such as mirror exposure and clothing practice (26), were reviewed for cultural sensitivity. Mirror exposure conducted privately at home does not require modification, as it is fully consistent with Islamic norms of modesty. However, adaptations are necessary for clothing-related and social exposure tasks, which are adjusted to align with religious beliefs and modesty expectations. These can take place in all-women environments or other settings consistent with Islamic principles that permit women to be uncovered in the presence of their “mahram” (close male relatives), other women, or when in private (Qur’an 24:31). Examples include women-only gyms, gender-segregated social settings, or modest swimwear when appropriate. These exercises address both self- and social-related body avoidance, helping patients gradually tolerate being seen by others in acceptable contexts. These adjustments preserve fidelity to the treatment’s mechanisms, reducing avoidance and weakening over-evaluation (26), while flexibly adapting the form of these procedures.

Adaptations related to modesty norms were unavoidable in this context: for veiled women, requiring uncovering in the presence of men would have disrupted treatment and violated religious and cultural norms. Body-image procedures were accordingly adapted in form (e.g., context and setting of exposure) while preserving their function of reducing avoidance and over-evaluation of shape and weight.

## 3 Discussion

This present paper synthesizes key insights from the cultural and clinical adaptation of CBT-E in the Middle East. This contributes to the broader effort to globalize evidence-based treatments for eating disorders, which remain largely Western-centric in their assumptions and evidence base (43–45).

The adaptation process described here was neither linear nor uniformly accepted. Implementation occurred in the context of substantial systemic and clinical barriers, including the absence of specialized inpatient or residential services, the need to manage severe medical risk within outpatient settings, and persistent workforce shortages and clinician turnover. The introduction of CBT-E was additionally met with professional skepticism within the local mental-health community. Its structured procedures were at times misinterpreted as rigidity or coercion, reflecting broader tensions that often emerge when evidence-based, manualized treatments are introduced into settings historically oriented toward eclectic or non-directive therapeutic traditions.

Ethical and relational considerations were an integral part of implementation, particularly at the intersection of medical care, family involvement, and religious practice. In many cases, family and religious frameworks functioned as supportive resources that facilitated engagement and reduced guilt around treatment decisions, including fasting abstinence during Ramadan. However, discussions were not always straightforward. When patients chose to continue fasting despite medical advice, their decisions were respected, and care shifted toward harm minimization through dietetic support, medical monitoring, and ongoing collaborative review. Religious authorities were consulted only at a preparatory level to clarify established exemptions for illness and did not participate in individual treatment decisions. Family involvement, when present, was voluntary and time-limited, but often required active boundary-setting and ongoing clinical work to ensure that support did not drift into control, while preserving patient autonomy and responsibility for change. Addressing these concerns required sustained psychoeducation, dialogue, and supervision. Given these structural inequities and the region’s distinct clinical and cultural realities, cultural adaptation was not merely useful. It was an ethically necessary step toward equitable implementation (43, 44). Embedding CBT-E within local cultural and religious frameworks, without altering its structure, bridges the gap between Western evidence and regional clinical realities. Without culturally sensitive delivery, diverse and underserved communities, including those in the Middle East, remain unable to fully benefit from treatments that constitute the global standard of care (43, 44).

Within this context, the present work contributes to decolonizing evidence-based practice and expanding inclusivity within global eating-disorder care (45, 46).

## 4 Conclusion

In conclusion, although eating disorders are present and clinically significant in the MENA, yet there remains a striking gap between treatment demand and the availability of culturally appropriate specialized, evidence-based care (4). Implementing CBT-E with culturally sensitive adaptations, suggests that such modifications can preserve treatment fidelity while enhancing relevance, engagement, and trust (20, 43, 44). The insights presented here were informed by accumulated clinical experience and routine service-level monitoring conducted as part of quality-improvement activities within our service, rather than a pre-specified research protocol. In parallel, a formal effectiveness study is currently underway to systematically evaluate CBT-E outcomes in this setting.

The present implementation-focused paper provides a foundation for future cultural adaptations by outlining transferable principles: maintaining core CBT-E mechanisms, adapting delivery to context, involving families selectively and collaboratively, and integrating religious practices pragmatically to enhance adherence across settings.

Such adaptation is not a departure from evidence-based practice but a necessary evolution of it, one that bridges scientific rigor with the lived realities of diverse populations (45). When guided by collaboration and contextual awareness, these adapted interventions have the potential to close regional treatment gaps and extend the reach, accessibility, and integrity of global evidence-based care (43, 44). Looking ahead, expanding Arabic-language resources (20, 30), strengthening regional training and supervision systems (20, 30), and building coordinated regional research and advocacy efforts (4, 5, 20, 45, 46) will be key to ensuring that culturally attuned, evidence-based treatments become accessible and sustainable across the Arab world (43, 44).

## Data Availability

The original contributions presented in the study are included in the article/**Supplementary Material**. Further inquiries can be directed to the corresponding author.
