# Supplementary material for: Adapting CBT-E for the Middle East: addressing regional gaps in eating-disorder treatment
Source: Front Psychiatry. 2026 May 12;17:1761708. doi: 10.3389/fpsyt.2026.1761708 (PMC13202229; doi:10.3389/fpsyt.2026.1761708)
Supplement: Supplementary file 1 [file Table1.docx]

**Supplementary Material**

**AI prompts used:**

**Initial Prompt:**
“Please help me edit my own written manuscript text for clarity, flow, and academic language. Do not introduce new ideas, concepts, interpretations, or scientific content. All arguments, structure, and conceptual work were developed by me; I only need assistance improving readability.”

**Final Prompt:**
“Please refine the final draft for coherence, grammar, and consistency of style while keeping all original concepts, interpretations, and scientific content exactly as written.”
